# Biology of GD2 ganglioside: implications for cancer immunotherapy

**DOI:** 10.3389/fphar.2023.1249929

**Published:** 2023-08-21

**Authors:** Pierre Machy, Erwan Mortier, Stéphane Birklé

**Affiliations:** Nantes Université, Univ Angers, INSERM, CNRS, CRCI2NA, Nantes, France

**Keywords:** ganglioside, GD2, cancer, immunotherapy, antibody, CAR, vaccine

## Abstract

Part of the broader glycosphingolipid family, gangliosides are composed of a ceramide bound to a sialic acid-containing glycan chain, and locate at the plasma membrane. Gangliosides are produced through sequential steps of glycosylation and sialylation. This diversity of composition is reflected in differences in expression patterns and functions of the various gangliosides. Ganglioside GD2 designates different subspecies following a basic structure containing three carbohydrate residues and two sialic acids. GD2 expression, usually restrained to limited tissues, is frequently altered in various neuroectoderm-derived cancers. While GD2 is of evident interest, its glycolipid nature has rendered research challenging. Physiological GD2 expression has been linked to developmental processes. Passing this stage, varying levels of GD2, physiologically expressed mainly in the central nervous system, affect composition and formation of membrane microdomains involved in surface receptor signaling. Overexpressed in cancer, GD2 has been shown to enhance cell survival and invasion. Furthermore, binding of antibodies leads to immune-independent cell death mechanisms. In addition, GD2 contributes to T-cell dysfunction, and functions as an immune checkpoint. Given the cancer-associated functions, GD2 has been a source of interest for immunotherapy. As a potential biomarker, methods are being developed to quantify GD2 from patients’ samples. In addition, various therapeutic strategies are tested. Based on initial success with antibodies, derivates such as bispecific antibodies and immunocytokines have been developed, engaging patient immune system. Cytotoxic effectors or payloads may be redirected based on anti-GD2 antibodies. Finally, vaccines can be used to mount an immune response in patients. We review here the pertinent biological information on GD2 which may be of use for optimizing current immunotherapeutic strategies.

## 1 Introduction

**FIGURE 1:**
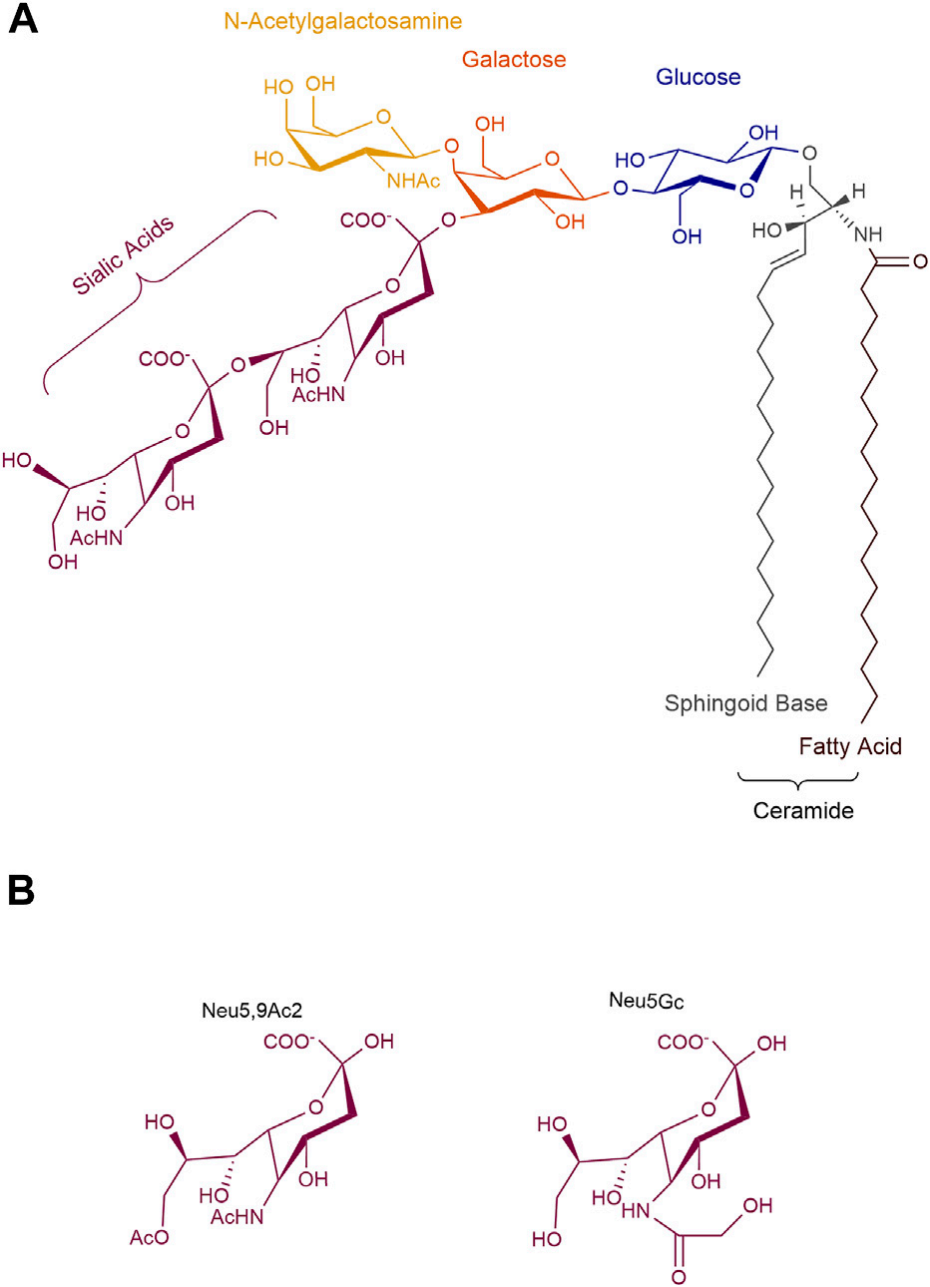
Structure of ganglioside GD2. **(A)** GD2 is an amphiphilic molecule that combines a hydrophobic ceramide to a hydrophilic oligosaccharide, containing two N-acetyl neuraminic acids. **(B)** Natural substitutions of sialic acids in GD2. Neu5Ac, N-acetyl neuraminic; Neu5Gc, N-glycolyl neuraminic acid; Neu5,9Ac2, 9-O-acetyl-5-N-acetyl neuraminic acid.

Although GD2-specific immunotherapies have shown clinical successes, our understanding of the biology of GD2, both in normal development and tumorigenesis, remains insufficient. Improving insufficient knowledge would make it easier to optimize anti-GD2 immunotherapies in a more informed and logical manner. GD2 ganglioside is a sialic acid-containing glycosphingolipid, whose structure is characterized by two distinct portions with different physicochemical properties. It is composed of a hydrophobic ceramide and a hydrophilic oligosaccharide containing one or more negatively charged sialic acids. Thus, gangliosides are amphiphilic molecules that have the ability to establish both hydrophilic and hydrophobic interactions. The ceramide, a sphingoid base linked to a fatty acid, interacts with other membrane lipids and allows GD2 to be tightly anchored to the cell surface. This ceramide tail is generally shared by all other gangliosides species. The oligosaccharide head is oriented towards the extracellular environment and interacts, via mild hydrophilic bonds, with neighboring membrane molecules or extracellular ligands. The term ganglioside GD2 is based on Svennerholm’s nomenclature (Svennerholm, 1963), where G stands for ganglioside, D for the number of sialic acid residues, and 2 corresponds to the order of its migration on thin-layer chromatograph. According to the IUPAC-IUBMB nomenclature, GD2 is named II^3^Neu5Ac_2_-Gg_3_-Cer, where the Roman number indicates the position of the sugar residue to which the sialic acid is linked considering the glucose in first position, the exponent Arabic number stands for the linkage position, the index Arabic number is for the number of sialic acids, and Gg3 indicates the following specific ganglio trisaccharide sequence: β-GalNAc-(1-4)β-Gal-(1-4)β-Glc-(1-1) (Chester, 1998). Thus, the chemical structure of human GD2 is β-GalNAc-(1–4)[α-Neu5Ac-(2-8)-α-Neu5Ac-(2–3)]β-Gal-(1-4)β-Glc-(1-1)Ceramide (Figure 1). Interestingly, GD2 has a universal base structure in all known species in contrast to proteins with variable homology between human and nonhuman species. However, the term ganglioside GD2 actually identifies a mixture of different GD2 subspecies with either different ceramides or sialic acids. These structural modifications impact both the biological and immunological properties of GD2 molecules as we will review here.

## 2 Biological significance of GD2 structural diversity

As described above, GD2 ganglioside is an amphiphilic molecule that combines a hydrophobic ceramide to a hydrophilic oligosaccharide, containing two negatively charged sialic acids at most physiological pH values (Figure 1). Variations in GD2’s oligosaccharide moiety mostly occur on the sialic acids, and are detected either by classical chromatographic techniques combined with antibody staining (Diaz et al., 2009), or, by combination of chromatography coupled with mass spectrometry analysis with higher sensitivity (Zarei et al., 2010). For example, 9-O-acetylated sialic acids on GD2 have been detected (Fleurence et al., 2017). This modification occurs during GD2 biosynthesis. The O-acetyl group is added by an O-acetyltransferase, possibly CASD1 (Baumann et al., 2015), to the carbon 7 of the terminal α2-8 linked sialic acid residue. This acetyl group further migrates to carbon 9 when exposed to higher pH (Fleurence et al., 2017). While the addition of the O-acetyl group on the terminal sialic acid of GD2 decreases polarity and hydrophobicity of the gangliosides, it does not affect general conformation: O-acetyl GD2 (OAcGD2) can be detected by most anti-GD2 monoclonal antibodies (Ye and Cheung, 1992; Fleurence et al., 2017). O-Acetylated gangliosides are often found in developping tissues and are regarded as onco-fetal antigens present on different tumors (Kohla et al., 2002), representing amounts up to 50% that of GD2 (Fleurence et al., 2017). As such, this GD2 subspecies, with more restricted normal expression than GD2 (see below) is of the most therapeutical significance.

The main sialic acid residue found in human ganglioside is N-acetylneuraminic acid (Neu5Ac), as an exon of the CMAH gene, encoding for the cytidine monophosphate-*N*-acetyl-neuraminic acid hydroxylase, the enzyme responsible for the synthesis of Neu5Gc is deleted in humans (Bashir et al., 2020). Another sialic acid variant is uses N-glycolylneuramic acid (Neu5Gc), usually observed in non-human mammals such as mice, and occasionally incorporated in human gangliosides from the diet through the consumption of red meat (Varki, 2009; Bashir et al., 2020). The exogenous incorporation is particularly seen in cancer cells due to their increased metabolism and induction of the sialin sialic acid transporter by hypoxia (Labrada et al., 2018). The presence of Neu5Gc leads to the development of varying levels of antibodies, which can lead to chronic inflammation and exacerbate cancer in mice models (Hedlund et al., 2008; Bashir et al., 2020)**.** Noteworthy, Neu5Gc-containing GD2 gangliosides have been described (Nishio and Furukawa, 2004), which could affect the affinity of anti-GD2 antibodies which binds in part to the terminal sialic acid (Ahmed et al., 2013), to limited effect due to ganglioside recycling and the minimal proportion these modified gangliosides represent (Nishio and Furukawa, 2004). This might represent, however, an opportunity to increase immunogenicity of GD2 gangliosides in vaccination efforts.

Disialogangliosides are also prone to form lactones at low pH. Lactones are cyclic esters of hydroxycarboxylic acids, formed through intramolecular esterification. GD3-lactone was identified in mouse brain (Gross et al., 1980) and human melanoma cells (Kawashima et al., 1994). However, no such report exists for GD2-lactone. Nonetheless, synthetic lactonization of GD2 was described, and demonstrated to possess increased immunogenicity. GD2-lactones induced an active humoral response in vaccination efforts, producing superior results compared to native GD2 (Ragupathi et al., 2003).

Structural heterogeneity is also found in the ceramide moiety. This can consist of different fatty acids and/or sphingoid bases. The latter consist of sphinganine, sphingosine, and phytosphingosine of different chain lengths, which can be further O-acetylated (Pruett et al., 2008; Hama, 2010). The most common sphingoid base found in human gangliosides is sphingosine (Ando and Yu, 1984; Sarbu et al., 2021), but GD2 ceramide can also include eicosphingosine, sphinganine, or phytosphingosine (Ando and Yu, 1984; Colsch et al., 2011; Merrill, 2011; Sibille et al., 2016). In addition, fatty acids can be unsaturated, saturated, oxygenated (Ladisch et al., 1989), and of different lengths, ranging from C16 to C24 (Ando and Yu, 1984; Sjoberg et al., 1992; Serb et al., 2009; Balis et al., 2020; Fabris et al., 2021). The functional consequences of the heterogeneities of the ceramide tail remain largely unknown. There are some indications that the lipid anchor composition determines ganglioside insertion into glycolipid-enriched microdomains through hydrophobic bonds with phospholipids, cholesterol, and glycosylphosphatidylinositol-anchored proteins (Furukawa et al., 2012). Within these microdomains, gangliosides interact with signaling molecules including receptor tyrosine kinases (Furukawa et al., 2012). Changes in the composition of the ceramide tail can thus result in the modification of plasma membrane fluidity and consequently in the deregulation of cellular signals. Furthermore, GD2 with shorter fatty acid chain (16 or 18 carbons) are more likely to be shed in the extracellular environment than longer chain GD2, in which they will be able to produce effects on bystander cells, including immunosuppression (Li and Ladisch, 1991; Ladisch et al., 1994). As another example, both fatty acid chain length and sphingoid structure influence the immunogenicity of glycosphingolipids, and thus, are of particular relevance to GD2 vaccination efforts (Okuda et al., 2019; Okuda, 2021). The ceramide moiety can also mask the receptor function of the oligosaccharide head through interaction with membrane cholesterol (Kolter, 2012). This observation raises inquiries about the asymmetrical distribution of glycosphingolipids in cellular membranes and tissue as GD2 detection and targeting is mediated by the oligosaccharide head. Indeed, ganglioside profiling should take into account both the oligosaccharide and the ceramide moieties.

## 3 GD2 biosynthesis

While the heterogeneity of the lipid tail results from the ceramide biosynthesis at the endoplasmic reticulum (Mandon et al., 1992; Sandhoff et al., 2018), the structural diversity of GD2 ganglioside oligosaccharide is generated within the Golgi apparatus as thoroughly reviewed elsewhere (Sandhoff et al., 2018; Sandhoff and Sandhoff, 2018). Intracellular synthesis of GD2 ganglioside begins with formation of the ceramide core. The synthesis of the sphingoid base is initiated by the condensation of a coenzyme A-activated fatty acid with L-serine, catalyzed by the serine palmitoyltransferase (SPT), to form 3-keto sphinganine (Ikushiro and Hayashi, 2011). Although palmitoyl CoA (16C) is the main substrate, SPT can also metabolize other acyl-CoAs, from C14 to C18, forming a variety of long-chain bases that differ by structure and function. In fact, SPT consists of three different core subunits (SPTLC1, SPTLC2, and SPTLC3) that condition the substrate specificity (Sandhoff et al., 2018). As an example, SPLTC3 induces a shift towards myristoyl-CoA (14C) and with the addition of serine, it produces sphingoid bases 16, 18 or 20 carbon long (Sandhoff et al., 2018).

The next step is the reduction of 3-keto sphinganine by 3-keto sphinganine reductase followed by acylation of sphinganine to hydroceramides of different chain lengths (Ikushiro and Hayashi, 2011). Six isoforms of ceramide synthase have been described and determine preferences for fatty acids of different chain length (Cingolani et al., 2016). At this stage, dihydroceramides can be dehydrogenated to ceramide by the dihydroceramide desaturases des1 (Fabrias et al., 2012), or hydroxylated to phytoceramides by des2 (Sandhoff et al., 2018).

Ceramides are then transported to the early Golgi apparatus vesicular transport or protein-bound transfer (Hanada et al., 2009; Sandhoff et al., 2018).

**FIGURE 2:**
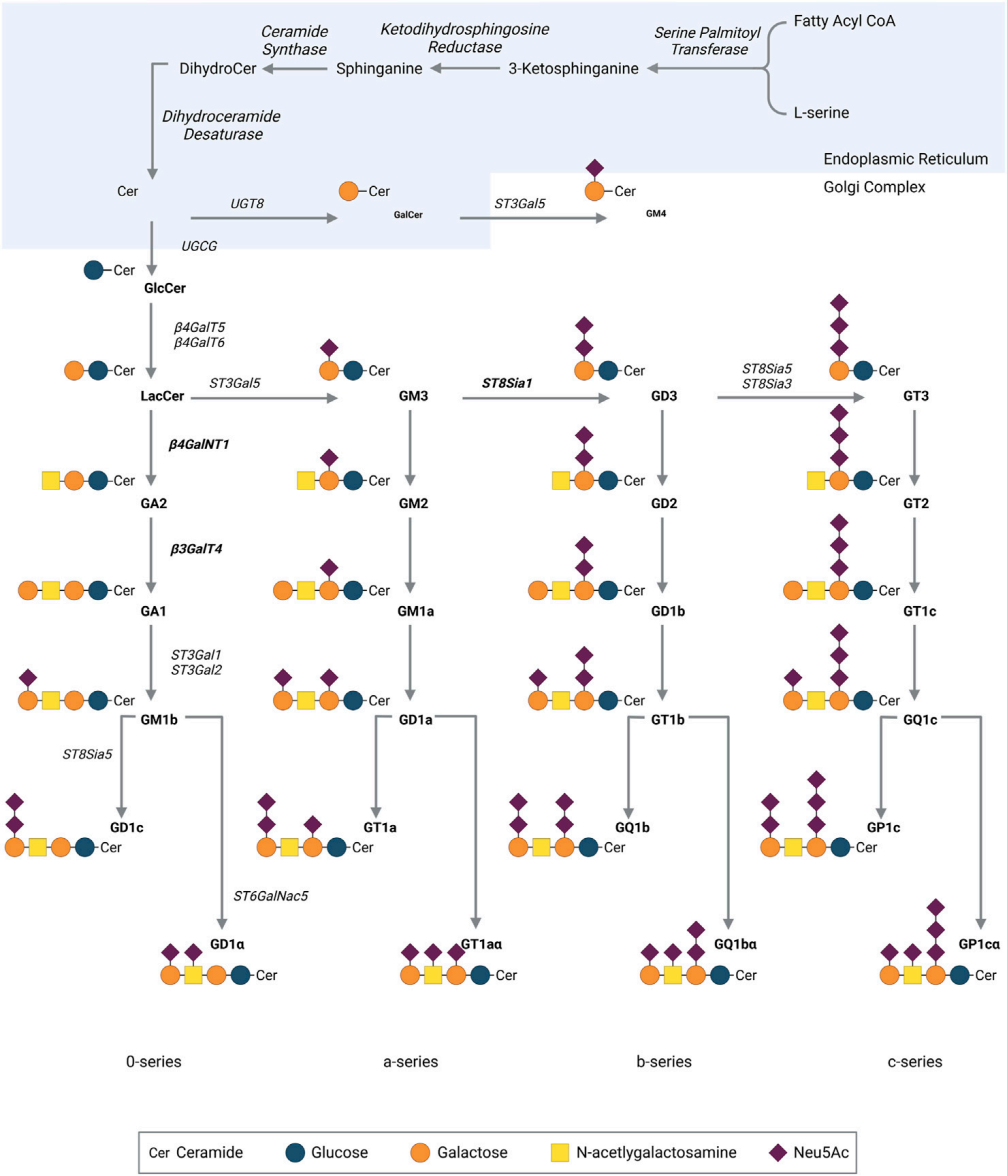
Pathway for ganglioside biosynthesis. Cer, ceramide, N-acylsphingosine; Lac-Cer, lactosyl ceramide; Gal, galactose; GalNAc, N-acetylgalactosamine; Glc, glucose; Sia, sialic acid; ST, sialyltransferase; GalT, galactosyltransferase; GalNT, N-acetyl-galactosaminyltransferase; GlcT, glucosyltransferase. Neu5Ac, N-acetyl neuraminic acid. Created with BioRender.com.

On the early Golgi’s cytosolic leaflet, addition of glucose to ceramides, mediated by UDP-glucose-ceramide-glucosyltransferase, produces glucosylceramide β-Glc-(1-1) Ceramide (GlcCer). GlcCer is then translocated to the luminal leaflet and the biosynthesis of the oligosaccharide moiety is synthesized in the Golgi apparatus by the sequential action of different glycosyltransferases (GT) and sialyltransferases (ST) (Figure 2). The sugar donor groups used in ganglioside synthesis are nucleotide sugars: UDP-saccharides for the sugar backbone, and CMP-sialic acid for the sialylations. Starting from GlcCer, the addition of a sialic acid by GM3 synthase (ST3 GAL 5, ST3 β-galactoside α-2,3-sialyltransferase 5) forms GM3 (Figure 2) (Ishii et al., 1998)**.** Thereafter, GM3 can be converted by GD3 synthase (ST8SIA1, ST8 α-N-acetylneuraminate α-2,8-sialytransferase 1) to form GD3, the key precursor to GD2 (Nagata et al., 1992). A final sialic acid added to GD3 by ST8Sia3 or ST8sia5 will form GT3. Downstream of these three precursors, gangliosides are synthesized by multienzyme hetero or homodimers, capable of using any of the precursor gangliosides as substrate. Using GD3, β4GalNT1 will link N-acetylgalactosamine (GalNAc) to the terminal galactose to form GD2. GD2 may in turn be used as substrate by β3GalT4, producing GD1b. This highlights GD2’s role as a metabolic intermediate (Figure 2).

GD2 ganglioside can be further modified by the addition of an O-acetyl group to the external sialic acid (Sjoberg et al., 1992). The O-acetylation reaction is catalyzed by Sialyl O-Acetyl Transferases (SOAT) and localized on membrane fractions and in the Golgi (Higa et al., 1989; Vandamme-Feldhaus and Schauer, 1998). With the help of recent screening methods, a single enzyme fitting all the necessary requirements was identified, encoded by the CASD1 (capsule structure1 domain containing 1) gene, whose overexpression in cellular models led to an increased expression of *O*-acetylated gangliosides (Arming et al., 2011). Additional work demonstrated that CASD1 transfers *O*-acetyl group on free sialic acids, and knock-out of CASD1 impedes cellular expression of *O*-acetylated gangliosides, underlining its essential role in this process (Baumann et al., 2015; Cavdarli et al., 2021).

While the acetyl donor group is universally identified as acetyl-CoA, there are divergent models regarding the formation process of O-acetyl-GD2 (OAcGD2). Conflicting studies suggest that the *O*-acetylation can either occur on C7 or C9 (Kamerling et al., 1987; Sjoberg et al., 1992). It is thought that the transfer primarily occurs on C7, with subsequent spontaneous migration to C9 possible under physiological pH (Varki and Diaz, 1984). Other reports show that OAcGD2 can be either obtained by the *O*-acetylation of GD2 by the SAOT or by the conversion of OAcGD3 by the β3GalT4 (Baumann et al., 2015; Cavdarli et al., 2020). Finally, according to initial models, the O-acetyl group is added on the terminal sialic acid, yet, recent data highlight the possibility of inner sialic acids’ acetylation (Cavdarli et al., 2020). Noteworthy, none of these mechanisms are exclusive, and may take place simultaneously with different importance. Interestingly, O-acetylation of sialic acids diminishes affinity of neuraminidases, which might consequently hamper the first step of ganglioside degradation (Hunter et al., 2018).

Surprisingly, OAcGD2 is concomitantly expressed with GD2 at the tumor cell surface (Alvarez-Rueda et al., 2011). This observation suggests another point of control in OAcGD2 biosynthesis. There are some indications that the synthesis of OAcGD2 can be regulated by the quantity of acetyl-CoA concentrations within the Golgi apparatus (Higa et al., 1989). This mechanism does not further exclude a possible turnover of O-acetyl esters bound to sialic acids of gangliosides controlled by sialate-O-acetylesterases (SIAE) (Butor et al., 1993). Thus, the expression of OAcGD2 in a cell type may be the result of the conjunction of, at least, three parameters: the balance between two enzymatic systems, SAOT and SIAE; the activity of the GM2/GD2 synthase that synthesizes OAcGD2 from OAcGD3; and the activity of GM1/GD1b synthase that forms GD1b from GD2 (Figure 2). Given the complexity of this biosynthetic model, the clarification of the mechanisms that regulate the expression of O-acetyl-GD2 remains challenging. At the end of the biosynthetic process, gangliosides are transported through vesicular transport on the surface of secretory vesicles to be expressed on the membrane.

Once at the cell surface membrane, GD2 ganglioside can be internalized. The kinetics of GD2 internalization have been reported in several studies after binding to specific antibodies (Wargalla and Reisfeld, 1989; Buhtoiarov et al., 2011; Tibbetts et al., 2022). The phenomena of GD2 internalization at the cell surface has several practical implications for designing anti-GD2 immunotherapies. For example, anti-GD2 immunotoxins have been designed and demonstrated some efficacy against GD2-expressing tumor cells *in vitro* and *in vivo* (Wargalla and Reisfeld, 1989; Mujoo et al., 1991). On the other hand, anti-GD2 internalization can also represent a mechanism for immunotherapy escape (Tibbetts et al., 2022). Yet, the mechanisms by which this occurs are not well understood. Studies performed with other ganglioside species suggest the existence of different endocytic processes such as clathrin- or caveolin-dependent mechanisms and autophagy (Sandhoff et al., 2018). Once in the early endosome, gangliosides can follow different pathways. Of note, the saturation of the ceramide acyl chain was reported to influence trafficking fate of gangliosides, with unsaturated acyl chain promoting recycling (Chinnapen et al., 2012). Ganglioside catabolism starts with the removal of sialic acids. This process is carried out by membrane-bound sialidases mainly located in the late endosomes. Once GD2 ganglioside is converted to its monosialylated GM2 equivalent, the terminal N-acetylgalactosamine is then removed by hexosaminidase, forming GM3. The final sialic acid is detached by sialidases and SAP-B to produce LacCer. β-galactosidases with their cofactors will produce GlcCer, and then ceramides under the influence of β-glucosidases with SAP-C. Finally, ceramidases and SAP-D split ceramides into sphingoid bases and fatty acids. Starting from GlcCer, catabolic fragments such as LacCer, ceramides or sphingosine can leave the lysosome to be used in new metabolic reactions, constituting salvage pathways (Tettamanti et al., 2003). To avoid catabolism, some gangliosides can be directly recycled back to the plasma membrane, and others can go through “direct glycosylation”, in which they are reintegrated in the Golgi and follow later stages of the biosynthesis pathway (Yu et al., 2008).

While glycosidases play a significant role in the breakdown of gangliosides, it has long been believed that the expression of cellular gangliosides primarily depends on the biosynthetic rather than catabolic processes. Essentially, in order to enhance the presence of a specific ganglioside, the cell needs to increase the enzyme responsible for its synthesis and/or reduce the activity of the enzyme that utilizes this ganglioside as its substrate (Bieberich and Yu, 1999; Mabe et al., 2022)**.** As a consequence, it is difficult to modulate the expression of a single ganglioside species at the enzymatic level.

## 4 Regulation of GD2 expression

As will be seen in further details later in this work, there is a tissue- and age-dependent restriction of GD2 expression (Svennerholm et al., 1991; Lammie et al., 1993; Huang et al., 2022). GD2 biosynthesis is mostly regulated by the activity of different glycosyltransferases, with emphasis on ST8SIA1, B4GALNT1 & B3GALT4 (Sorokin et al., 2020; Sha et al., 2022), and is controlled epigenetically, transcriptionally, and post-transcriptionally.

Common to the transcriptional regulation of all aforementioned genes, and ganglioside glycosyltransferase in general, is their lack of TATA- and CCAAT-boxes, or any known core promoter elements (Kasprowicz et al., 2022). GC-rich boxes were however observed in the promoters (Yu et al., 2004). These features correspond to housekeeping genes, genes under little regulation. This apparent lack of regulation underlines the importance of another level of gene expression regulation: epigenetic modifications (Itokazu et al., 2017). Epigenetic regulation of ganglioside patterns is seen during development in physiological conditions (Tsai and Yu, 2014; Itokazu et al., 2017). In cancers expressing low levels of GD2 such as Ewing sarcoma and some neuroblastomas (NB), inhibition of silencing epigenetic modifications (detailed later in this work) increases significantly GD2 expression (Kroesen et al., 2016; Kailayangiri et al., 2019; van den Bijgaart et al., 2019; Mabe et al., 2022). However, the same treatment on healthy counterpart cells did not lead to increased GD2 (Mabe et al., 2022). This suggests additional levels of regulation differentiating normal from aberrant cells. This distinction can be a result of varying transcriptional regulations in the three genes mainly responsible for GD2 expression (Sorokin et al., 2020; Sha et al., 2022). This additionally means that GD2 can be a useful stage-specific marker molecule of developing cells.

ST8SIA1, the gene coding for the GD3 synthase is essential to GD2 expression through the production of GD3, precursor and bottleneck to all b-series ganglioside synthesis (Freischütz et al., 1995; Tsai and Yu, 2014; Mabe et al., 2022)**.** The gene possesses multiple initiation sites in the human promoter region. However, alternative transcripts have rarely been observed, with weak expression in some cancers with no particular effect described (Kasprowicz et al., 2022). Of interest, the promoter region contains binding sites for transcription factors such as c-Ets-1, CREB, AP-1, and NF-κB (Kasprowicz et al., 2022)**,** associated with cancers and inflammation. In addition, work on rat PC12 cells showed that alternative use of transcription factors could differentiate between high- and low-ST8SIA expressing cells (Yu et al., 2008). This implies a potential modulation of GD2 expression by microenvironmental cues. A negative control region has similarly been described, but data is lacking regarding its relevance (Furukawa et al., 2003).

B4GALNT1, the gene coding for GD2/GM2 synthase, is under complex regulation. There are three promoters for this gene, corresponding to three transcription start sites and three different exons. In the different promoters, distinct transcription factor binding sites are present, implying differential regulation. Furthermore, an enhancer for two promoters, as well as a silencer for the third have been described (Yu et al., 2004). Alternative use of promoters, enhancers and silencers may regulate cell-specific expression.

B3GALT4, the gene coding for the GD1b/GM1 synthase similarly possesses multiple promoters with associated transcription factor binding sites, and transcription start sites (Al-Obaide et al., 2015). Tissue or cell specificity was noted for promoter use and transcription factor binding sites. Alternative promoter or transcription factor use may be a mechanism leading to higher GD2 expression (Yang et al., 2022). In addition, the promoter regions contain cancer-associated transcription factors binding sites, linking tumorigenesis to ganglioside expression (Al-Obaide et al., 2015).

In a final regulation layer, glycosyltransferases form complexes in their respective compartments. Multiple enzymes can form heterodimers in addition to homodimers: ST8Sia1/ST3Gal5, ST8Sia1/B4GalNT1, B4GalNT1/B3GalT4 (Yu et al., 2004). Dimer formation is influenced by enzyme composition: overexpression of either ST8Sia1 or B4GalNT1 in normal cells lead to increased ST8Sia1/B4GalNT1 with concomitant decrease of dimers using other partners (Bieberich et al., 2002). While dimerized, ST8Sia1 & B4GalNT1 transactivate each other to produce GD2 from GD3 (Bieberich et al., 2002). These mechanisms potentiate simple enzyme up- or downregulation and lead to increased surface GD2. In this model, additional regulation of GD2 synthesis may be mediated by post-translational modifications of glycosyltransferases, such as N-glycosylation that has been described to affect enzyme cellular localization (Bieberich et al., 2000). However, limited details concerning the specifics of these modifications, potentially druggable, are available in the literature (Yu et al., 2004; Yang et al., 2022).

## 5 GD2 expression in normal tissues

GD2 expression has been reported in the brain to varying degrees according to developmental stages (Svennerholm et al., 1991; van den Bijgaart et al., 2019). While GD2 represents 5%–7% of brain gangliosides during gestation, the proportion progressively declines until it reaches 2% in adult brains (Svennerholm et al., 1991; van den Bijgaart et al., 2019). In comparison, its precursor, GD3, maintains a stable proportion of 5% of brain gangliosides through development (Svennerholm et al., 1989). This underlies development-dependent restriction of GD2 expression, most likely in favor of more complex gangliosides highly expressed in adult brains such as downstream GD1 (Svennerholm et al., 1989; Sipione et al., 2020). After birth, GD2 expression has been reported in various brain zones such as the hypophysis, the hypothalamus, the hippocampus, and the midbrain (Svennerholm et al., 1994; Yuki et al., 1997; Colsch et al., 2011). Concerning nervous tissues outside the brain, GD2 expression has been detected on the spinal cord, cauda equina, and the peripheral nerves (Svennerholm et al., 1994; Yuki et al., 1997; Colsch et al., 2011). GD2 expression was additionally detected in the skin (Hersey et al., 1988), and in human prostate cells (Shiraishi et al., 1988).

Regarding cellular expression, surface GD2 has been detected in various populations. Melanocytes, the pigmental cells of the skin, have also been shown to express GD2, in line with their neuroectodermic origin (Svennerholm et al., 1994). Noteworthy, expression of GD2 has been detected on T cells activated artificially or in disease (Hersey and Jamal, 1989; Villanueva-Cabello et al., 2015; Villanueva-Cabello and Martinez-Duncker, 2016). This may have an impact on GD2-targeting therapies that aim to engage the immune system, as T cells may be targeted as well, with particular relevance for GD2 CAR-T cells. Among immune populations, one report noted GD2 expression on B cells and reticular dendritic cells in lymph nodes of melanoma patients (Hersey and Jamal, 1989). More recently, GD2 was identified as a marker of mesenchymal stromal cells (MSC), be they from the bone marrow or umbilical cord (Martinez et al., 2007; Santilli et al., 2022). This point is of particular interest in the field of oncology, as MSC are often found in tumors with various pro-tumorigenic roles such as immune-inhibition and angiogenesis (Kidd et al., 2008; Lee and Hong, 2017). Targeting of tumors expressing GD2 may as such yield additional benefits in microenvironment regulation, although further studies are needed for validation on cancer-associated MSCs.

Of note, the O-acetylated form of GD2, OAcGD2, displays more restricted expression than GD2. Among 32 tissues tested by immunohistochemistry, OAcGD2 was only weakly detected in specific zones of few tissues. OAcGD2 was detected in the zona reticularis of the adrenal medulla, some macrophages of the bone marrow, the germinal centers of lymphoid follicles, as well as the Purkinje neurons and the gray matter of the dorsal horns (Alvarez-Rueda et al., 2011).

## 6 GD2 expression in cancer

Overexpression of GD2—significant increase in GD2 compared to healthy tissue–has been reported in various cancers (Tab.1) (Nazha et al., 2020). Interestingly, most of these tumors are pediatric cancers. As such, increased GD2 may reflect aberrant developmental processes, with cells less differentiated compared to healthy counterparts and not having completed the transition to expression of more complex gangliosides such as GD1 (Filbin and Monje, 2019). In this sense, it is intriguing to note that GD2 is increased specifically on breast cancer stem cells. Additionally, in several GD2-positive cancers, OAcGD2 is correspondingly overexpressed (Table 1). Combined with its more restricted normal expression and considering that anti-OAcGD2 antibodies have no cross-reactivity with GD2, targeting OAcGD2 may decrease on-target off-tumor anti-GD2 immunotherapy adverse effects in patients (Alvarez-Rueda et al., 2011; Terme et al., 2014).

**TABLE 1:** Tumors overexpressing GD2 and OAcGD2 gangliosides.

Cancer	% Tumors expressing GD2	% Tumors expressing OAcGD2
Neuroblastoma	95%—100% Wu et al. (1986); Kramer et al. (2001)	100% Alvarez-Rueda et al. (2011)
Osteosarcoma	85%–100% Roth et al. (2014); Poon et al. (2015); Dobrenkov et al. (2016)	-
Gliomas	80% Longee et al. (1991)	100% Fleurence et al. (2017)
Melanoma	25%—75% Hersey et al. (1988); Dobrenkov et al. (2016)	75% Alvarez-Rueda et al. (2011)
Small Cell Lung Cancer (SCLC)	50%—100% Cheresh et al. (1986b); Grant et al. (1996)	75% Alvarez-Rueda et al. (2011)
Ewing sarcoma	40%–100% Grant et al. (1996); Dobrenkov et al. (2016); Wingerter et al. (2021)	-
Soft tissue Sarcomas	93% (Depends on sarcoma) Chang et al. (1992)	-
Retinoblastoma	37%–100% Portoukalian et al. (1993); Sujjitjoon et al. (2021); Wang et al. (2022)	-
Breast Cancer	2%—35% (On Breast cancer stem cells) Battula et al. (2012)	On Stem cells Cheng et al. (2021)
Ovarian cancer	78% Galan et al. (2023)	-

GD2 overexpression can be further correlated with the upregulation of ST8SIA1 and B4GALNT1, or the downregulation of B3GALT4 (Sorokin et al., 2020; Yoshida et al., 2020; Kasprowicz et al., 2022; Mabe et al., 2022; Sha et al., 2022). So far, no mutation has been reported for these genes, and the specific causes of GD2 overexpression remain unclear.

At the plasma membrane level, GD2 has been identified as a lipid raft component (Sha et al., 2022). Lipid rafts are specialized, sphingolipid-enriched, dynamic microdomains within the membrane that can compartmentalize cellular processes. Within lipid rafts, signaling proteins such as tyrosine kinases can either be recruited or excluded, to regulate signal transduction (Mollinedo and Gajate, 2020). Several reports have demonstrated the role of GD2 in modulation of cell signaling. In NB cells, enrichment of GD2 has been observed in lipid rafts, and inhibition of GD2 synthesis through B3GALT4 silencing, leads to decreased rafts within cells (Sha et al., 2022).

While GD2 and OAcGD2 represent interesting targets for these multiple cancers, recent works suggest that GD2 expression is rather heterogeneous in tumors, including neuroblastoma (Schumacher-Kuckelkorn et al., 2017; Terzic et al., 2018; Dondero et al., 2021)**.** Several reports further suggest that GD2 expression level may predict anti-GD2 antibody therapeutic responses (Terzic et al., 2018; Hirabayashi et al., 2021; Theruvath et al., 2022). As such, studies on the mechanisms influencing GD2 tumor cell expression are required to optimize anti-GD2 therapies. Several parameters associated with the tumor microenvironment have been identified so far. For example, it has been noted that hypoxic conditions led to overexpression of the key enzyme to GD2 production, ST8SIA1, in colon tumor cell lines (Yin et al., 2010). Similarly, osteosarcoma spheroids had gradually increased GD2 expression over time, concurrent with cell growth and densification of the sphere causing a decrease in oxygen availability (Wiebel et al., 2021). Further studies are required to confirm the link between hypoxia and GD2 expression. Immunostimulatory cytokines were also tested on diverse cancer models for their capacity to enhance GD2 expression. The cytokines, IL-4, TNF-α and IFN-γ, all increased GD2 expression as a single agent or in combination with each other (Hoon et al., 1991a; Hoon et al., 1991b). TNF-α and IFN-γ are cytokines produced either by innate or adaptive immune cells (Castro et al., 2018; Josephs et al., 2018)**.** Therefore, it is of particular relevance to immunotherapies as immune cell activation may lead to increased target expression by bystander cells.

GD2 expression in tumors has also been studied in respect to the cellular intra-tumoral heterogeneity and tumor cell plasticity. A small subpopulation of cells within tumors with self-renewal capacities are named cancer stem cells (CSC). CSCs have been linked with resistance to conventional chemotherapy and radiotherapy, and thus, are believed to drive tumor progression and disease recurrence (Phi et al., 2018). Targeting CSCs is therefore important for limiting tumor spread and recurrence. Interestingly, the expression of GD2 has been evidenced in breast carcinoma CSCs (Battula et al., 2012; Liang et al., 2013)**.** In parallel, factors modulating the expression of GD2 in breast CSCs have been identified. In various models of triple negative cancer cell lines, oxidative stress, caused by disturbances in the normal redox state of cells, induced increased stemness in cells and as such increased percentages of GD2-positive cells (Jaggupilli et al., 2022). In addition, OAcGD2 expression was also confirmed in breast CSCs (Cheng et al., 2021).

Another source of intra-tumoral heterogeneity with regards to GD2 expression stems from plasticity. Cell plasticity allows tumor cells to change their phenotypic characteristics without necessitating further genetic mutations in response to environmental cues. For instance, in NB there is a more differentiated “noradrenergic” state, and a less-differentiated “mesenchymal” state (Boeva et al., 2017; van Groningen et al., 2017). When diagnosing human primary NBs, the noradrenergic state is found to be the predominant state in the tumor (Boeva et al., 2017; van Groningen et al., 2017), while the MES state may be more prevalent in relapse and metastatic disease according to studies on human tumors and mouse models (Boeva et al., 2017; van Groningen et al., 2019; McNerney et al., 2022). Interestingly, “mesenchymal” NB cells demonstrate lower GD2 expression level than “noradrenergic” NB cell lines, with greater resistance to anti-GD2 immunotherapy (Mabe et al., 2022; McNerney et al., 2022).

Given the emerging role of cancer plasticity in anti-GD2 immunotherapy, the development of strategies targeting the underlying mechanisms of plasticity may lead to durable responses. At the cellular level, epigenetic modification is a promising approach that allows regulation of glycosyltransferase gene expression (Kroesen et al., 2016; Kailayangiri et al., 2019; Mabe et al., 2022). Histones, the DNA packaging proteins, can be acetylated or methylated leading to changes in chromatin architecture that can increase or decrease gene expression through DNA accessibility. EZH2 is part of the PRC2 complex and is involved in histone methylation, such as the silencing modification of histone 3, H3K27me3. Use of EZH2 inhibitors in various potentially GD2-positive cancers such as neuroblastoma and Ewing sarcoma led to increased GD2 expression through upregulation of ST8SIA1, and in some cases B4GALNT1 (Kailayangiri et al., 2019; Mabe et al., 2022). Similarly, Tazemetostat, an inhibitor of EZH2, successfully altered the cellular state in “mesenchymal” NB. This reprogramming led to the activation of “noradrenergic” gene expression, including ST8SIA1, resulting in an increase in GD2 expression (Mabe et al., 2022).

In the same line, histone deacetylase (HDAC) catalyzes, amongst others, deacetylation of the activating modification H3K27ac. Use of HDAC inhibitors enhanced GD2 expression on neuroblastoma cells through upregulation of ST8SIA1 (Kroesen et al., 2016; van den Bijgaart et al., 2019). This effect could be further augmented through exogenous addition of sialic acids (van den Bijgaart et al., 2019).

Although both HDAC inhibitors and EZH2 inhibitors, have FDA-approval in different cancers (Mann et al., 2007; Straining and Eighmy, 2022), these treatments are nonspecific and lead to a general decrease of silencing marks or increase of activating marks. This causes up- and downregulation of numerous other genes and as such precautions are necessary in their use.

In a more technical approach, but particularly relevant to the use of 2D and 3D *in vitro* models, it has been demonstrated that cell confluency modulates GD2 expression, whether in monolayer or spheroid culture (Wiebel et al., 2021). If unchecked, this parameter may negatively impact data reproducibility. The mechanisms linking cell confluency and GD2 expression have not been described, but hypoxia and oxidative stress due to reduced nutrient may play a role. In addition, these microenvironmental stress factors may induce epigenetic rewiring, of particular importance for GD2 expression as mentioned above.

## 7 Functional aspects of GD2 ganglioside in cancer progression

As described above, GD2 is overexpressed in several cancer types. Nevertheless, comprehension of the biological consequences stemming from its overexpression have yet to be unequivocally determined. Several roles of GD2 have been suggested using different experimental approaches. For example, exogenous GD2 can be added in the culture media to be incorporated in cells’ plasma membranes (Li et al., 1996). Anti-GD2 blocking antibodies can also be used to disrupt GD2 interaction with other cell membrane components. Pharmacological depletion of the cellular gangliosides can be achieved using glucosylceramide synthase inhibitors such as PDMP (Inokuchi et al., 1990) or PPMP (Lee et al., 1999). Alternative methods consist in gene transfer (Yoshida et al., 2001) or gene editing (Bhat et al., 2023).

However, it should be noted that these experimental approaches have technical limitations. Because of the nature of GD2 as a metabolic intermediate in a sequential process, targeting the enzymes responsible for GD2 biosynthesis may result in a complex pattern of interference making it difficult to identify the molecular ganglioside species involved. For example, knocking out GD3 synthase will result in the accumulation of GD3 precursors and the disappearance of its downstream products. While GD2-blocking antibodies have the advantage of precisely identifying GD2, their different modes of actions such as antigen blocking, masking, clustering or internalization hinder understanding of the normal roles in cancer cells. Nonetheless, these different approaches have evidenced several biological effects of GD2 particularly relevant to tumor progression and immunosuppression as discussed below.

Among first studies were conflicting reports pointing to a link between GD2 expression and either adhesive or anti-adhesive properties. Regarding anti-adhesive properties, one work has shown that GD2 is a cellular receptor for tenascin-C, an extracellular matrix protein found in embryonic, inflamed, or cancerous tissues, leading to cell detachment from fibronectin through inhibition of focal adhesion (Probstmeier and Pesheva, 1999). A study using osteosarcoma cells similarly showed that GD2 overexpression led to increased cell motility (Shibuya et al., 2012)**.** In that context, GD2 expression has been linked with the EMT process in bladder cancer which could be reversed upon inhibition of GD2 synthesis (Vantaku et al., 2017)**.** Contrariwise, multiple work based on cancerous models such as melanoma and neuroblastoma indicate a role in adhesion. Acting as a co-receptor for integrins in binding to various extracellular matrix proteins such as fibronectin, vitronectin and collagen, blocking of GD2 can inhibit adhesion (Cheresh et al., 1986a; 1987; Yesmin et al., 2021)**.** Altogether, these observations suggest that effects of GD2 on adhesion are context dependent, with a possible dependence on integrin type (Furukawa et al., 2012)**.**

It is interesting to note in either case, GD2 is associated with integrins. In this line, different works have demonstrated localization of GD2 in adhesion plaques and presence of trimeric complexes of GD2, Focal Adhesion Kinase (FAK) and integrins, (Cheresh et al., 1984; Aixinjueluo et al., 2005). In SCLC cells, GD2-specific antibodies disrupted the GD2/integrin complexes at the tumor cell surface resulting in the dephosphorylation of FAK and subsequent anoikis. Inversely, overexpression of GM2/GD2 synthase reduced anoikis in melanoma models. The association of GD2 with FAK/AKT signaling pathway were later reported by others in high-expressing GD2 melanoma cells (Yesmin et al., 2021), glioma cells (Iwasawa et al., 2018), prostate cancer cells (Nguyen et al., 2018; Xing et al., 2021), triple negative breast cancer cells (Nguyen et al., 2018), and osteosarcoma cells (Liu et al., 2014). FAK signal through the AKT/mTOR pathway to promote processes such as cell survival, cell motility, angiogenesis (Chuang et al., 2022)**.** In the context of cell attachment, FAK activation is a necessary step in the outside-in signaling mediated by integrins when bound to matrix proteins, and leads to pro-survival signaling (Mitra and Schlaepfer, 2006). In the context of cell detachment and motility, FAK activation promotes anchorage independent growth and consequently tumor cell survival during dissemination (Duxbury et al., 2004; Liu et al., 2008; Deng et al., 2021). As such, activation of FAK, possibly facilitated by its localization in the increased GD2-dependent lipid rafts, may be a link between the opposite adhesive/anti-adhesive roles of GD2 in different contexts. In this sense, another example of modulation of the tumor signaling pathways by GD2 was identified in breast cancer cells. In GD3 synthase transfected breast cancer cells, GD2 colocalized with the receptor tyrosine kinase cMET and was responsible for its constitutive activation, leading to increased proliferation through downstream MAPK signaling (Cazet et al., 2012)**.** This effect was blocked either by silencing GM2/GD2 synthase, or by anti-GD2 antibodies treatment (Cazet et al., 2012).

Both adhesive/anti-adhesive mechanisms link back to a positive correlation between GD2 expression and invasiveness in different context such as melanomas, osteosarcoma (Shibuya et al., 2012; Yesmin et al., 2021), gliomas (Iwasawa et al., 2018), triple negative breast cancer (TNBC) and prostate cancer (Nguyen et al., 2018; Xing et al., 2021). In this sense, anti-GD2 incubation decreased matrix-metalloproteinase 2 production in osteosarcoma (Liu et al., 2014).

Regarding other processes of cancer dissemination, overexpression of GD2/GM2 synthase in a melanoma model increased angiogenesis, potentially through a drastic increase in periostin production, a protein with implications in angiogenesis and migration (Huizer et al., 2020; Yoshida et al., 2020).

Other molecular alterations induced by anti-GD2 may be implicated in antigen-positive cells’ apoptosis. In different cancer context such as neuroblastoma and small cell lung cancer, incubation of anti-GD2 antibodies led to cell death without any immune mediator, through activation of the caspase-3 pathway inducing apoptosis (Yoshida et al., 2001; Kowalczyk et al., 2009; Tsao et al., 2015; Iwasawa et al., 2018). Interestingly, use of antibodies directed against O-acetyl GD2 similarly induced apoptosis in OAcGD2-expressing neuroblastoma cells through caspase-3 activation (Cochonneau et al., 2013).

Along the same lines, in reports based on neuroblastoma cell lines, incubation with anti-GD2 diminished Aurora kinase A and MYCN expression, while PHDLA1, P53 and c-jun’s were strengthened (Horwacik et al., 2015; Horwacik et al., 2013). Aurora kinase A is involved in cell proliferation and is crucial to complete mitosis. Aurora kinase A additionally protects from degradation MYCN, a major driver of oncogenesis in neuroblastoma. Inversely, aurora kinase A promotes tumor-suppressor p53 degradation. On the opposite, PHDLA1 is a tumor suppressor inhibiting AKT and Aurora kinase A, and is regulated by p53 (Chen et al., 2018). As such, incubation with anti-GD2 induces a shift from a tumor-promoting function characterized by increased Aurora A kinase expression, to a tumor-suppressing function characterized by increased PHDLA1, in line with the observed increased apoptosis. However, c-jun expression–regarded as a proto-oncogene–was similarly increased following anti-GD2 treatment. Interestingly, c-jun may rather favor apoptosis, as it mediates expression of pro-apoptotic factor following Jun kinase activation, as described after anti-GD2 incubation (Yoshida et al., 2002).

In addition, GD2 may be implicated with regulation other hallmark of cancer, such as cancer stem cells (CSC) and treatment resistance. Expressed on breast CSCs in TNBC, it has been reported that the genes associated with CSC properties such as SOX2, BCL11A, FOXC1, are in fact tightly regulated by ST8SIA1 (Nguyen et al., 2018). Still in TNBC, upregulation of ST8SIA1 was associated in patients with chemoresistance. Inhibition of ST8SIA1 enhanced the efficacy of the chemotherapy, concomitant with a suppression of the FAK/Akt/mTOR and Wnt/β-catenin pathways (Wan et al., 2021). Both of these reports may be linked by the fact that cancer stem cells generally display increased chemoresistance (Abdullah and Chow, 2013; Zhao, 2016).

Although unrelated to cancer functions, injection of anti-GD2 is often followed by allodynia, due to GD2 expression on peripheral nerves (Anghelescu et al., 2015). This was explained by antibody mediated activation of Src kinases cascading to activation of the NMDA-Receptor, involved in peripheral nerve sensitization. It is interesting to note that FAK kinase activation mediates among others Src kinase activation.

Apart from tumor-centric roles, GD2 might be involved in regulation of the tumor immune microenvironment. In gliomas, knock-out of ST8SIA1 increased the number, and activation status, of microglia/macrophages inside the tumor via reduced IL-6 and TGF-β1 (Zhang et al., 2021). Going further, the general changes in chemokine and cytokine production observed following the knock-out favored polarization towards the M1 phenotype rather than the M2-tumor promoting phenotype enriched in wild-type gliomas (Zhang et al., 2021). In neuroblastomas, based on overexpression of B3GALT4, forming GD1b from GD2, one report described enhanced recruitment of CD8^+^ T cells. The enzyme overexpression decreased GD2 quantity and consequently the formation of lipid rafts. In turn, this reduced c-Met signaling and downstream AKT/mTOR/IRF-1 pathway, with one finality being the increased expression of CXCL9 and CXCL10, responsible for cytotoxic T cell recruitment (Sha et al., 2022). It can thus be hypothesized that GD2 has a role in immune microenvironment suppression, both at the innate and adaptive level.

Like other glycosphingolipids, GD2 is in part shed from the plasma membrane, mostly as micelles or membrane vesicles (Ladisch et al., 1987; Li and Ladisch, 1991; Kong et al., 1998). Circulating GD2 can be measured in the serum of patients with GD2-expressing tumors, such as NB and retinoblastomas (Valentino et al., 1990; Portoukalian et al., 1993). Thus, it can be proposed as a companion diagnostics to anti-GD2 immunotherapies.

Shed gangliosides can be also incorporated in the plasma membrane of the neighboring cells (Chang et al., 1997), thus it is possible that GD2 can modulate tumor-bystander cell interactions. In this line, GD2-positive melanoma cells with increased cell growth, invasion, and adhesion secreted extracellular vesicles that could enhance properties of neighboring GD2 negative cells to similar levels (Yesmin et al., 2023). There are also several studies evidencing a role of GD2 in inhibition of T cell reactivity both *in vitro* and *in vivo* (Floutsis et al., 1989; Ladisch et al., 1992; Li et al., 1996). This diminished activity, evaluated through lymphoproliferative responses was valid for both non-specific mitogens such as PHA and soluble antigens such as toxoids (Ladisch et al., 1992). The effects on T cells, while significantly lower, were comparable to potent doses of cyclosporine A, an immunosuppressive agent (Li et al., 1996). Interestingly, shed GD2 was detected incorporated into T cells, and these T cells were significantly more apoptotic than GD2 negative ones. In addition, variations in immunosuppressive potential were observed depending on the ganglioside subspecies. Shorter fatty acid chains (C18 or less) were associated with increased immunosuppression for GD2 (Ladisch et al., 1994). Surprisingly, GD2 extracted from tumors were more immunosuppressive compared to healthy human brains’, which might imply enrichment of different GD2 subspecies in cancer than in physiological conditions (Ladisch et al., 1994). Other modifications modulating this property are modifications to sialic acids, with lactones less immunosuppressive than their classical counterparts, and hydroxylation of the fatty acid, displaying a similar trend (Ladisch et al., 1992; Ladisch et al., 1995). Similarly, using purified GD2, and inhibition of dendropoiesis, and dendritic cell activation by four-fold was shown (Shurin et al., 2001). This implies dysfunctions in the generation of possible adaptive immune responses to GD2 expressing cancers. On the contrary, incubation of NK cells with shed GD2 did not hinder cell cytotoxicity, nor their maturation (Ando et al., 1987; Grayson and Ladisch, 1992; Liu et al., 2022).

Finally, GD2 can also regulate direct tumor-immune cell interactions. In a recent study, GD2 blocking antibodies allowed the identification of GD2 as a ligand for the immune checkpoint receptor siglec-7, expressed on monocytes and NK cells (Ito et al., 2001; Theruvath et al., 2022). This finding arises on the observation that a Fc-dead anti-GD2 antibody enhances ADCP of GD2-positive tumor cells by macrophages in the absence of Fc binding or complement activation (Theruvath et al., 2022). The final demonstration was brought by evidencing the binding of recombinant siglec-7 onto GD2 gangliosides (Theruvath et al., 2022). Given these observations, similar work conducted on NK cells might yield interesting results.

## 8 GD2 as a target antigen for immunotherapies

### 8.1 First successes: monoclonal antibodies

The most representative therapeutic targeting of GD2, paving the way for all other forms immunotherapies, are monoclonal antibodies. The murine IgG3 monoclonal antibody (mAb) 3F8, mediates tumor cell killing directly through GD2 binding, as mentioned earlier, and through antibody dependent cell cytotoxicity (ADCC), complement dependent cytotoxicity (CDC), as well as phagocytosis of intact GD2 positive cells (Munn and Cheung, 1989; Munn and Cheung, 1990; Kushner and Cheung, 1991). Phase II studies on patients with stage 4 neuroblastoma, 40% responded with in some cases nearly 50% of patients progression-free (Cheung et al., 1998a; Cheung et al., 1998b). These clinical studies confirmed the implication of ADCC, with both granulocytes and NK cells’ activation potential correlating positively with treatment outcome (Delgado et al., 2010; Cheung et al., 2012; Tarek et al., 2012).

Another murine antibody, 14G2a, IgG2a class-switched from the IgG3 14.18, was shown to enable ADCC and CDC (Mujoo et al., 1987; Mujoo et al., 1989). In Phase I studies in various contexts such as melanoma, neuroblastoma, and osteosarcoma clinical utility of 14G2a varied depending on the context, going from 25% of patients respondent, up to 66% in neuroblastoma, with two complete remissions and two partial remissions (Handgretinger et al., 1992; Saleh et al., 1992; Murray et al., 1994).

A similarity of 3F8 and 14G2a is that both of them are cross-reactive with NCAM, a neural adhesion molecule (Patel et al., 1989; Agrawal and Frankel, 2010). Affinity for GD2 however is a differentiating factor as 3F8 had higher affinity than 14G2a (Cheung et al., 2012).

In order to reduce production of human anti-mouse antibodies, efforts were carried out to reduce murine components of these antibodies (Handgretinger et al., 1992; Saleh et al., 1992; Murray et al., 1994; Cheung et al., 1998a; Cheung et al., 1998b).

Chimeric antigen ch14.18 was produced by fusing heavy and light chains of murine 14.18 (Gillies et al., 1989). Chimeric ch14.18 increased efficiency of ADCC (Mueller et al., 1990) and prolonged half-life (Uttenreuther-Fischer et al., 1995). Analysis on 11 years follow-up of the 1st large scale study demonstrated benefit on overall survival when compared to chemotherapy for maintenance in neuroblastoma (Simon et al., 2004; 2011; Perez Horta et al., 2016). Building up on the role of ADCC in treatment outcome, a phase III trial combining Ch14.18 with IL-2, GM-CSF and retinoic acid for neuroblastoma showed superiority to the standard of care chemotherapy considering both overall survival (73.2%) and progression free survival (56.6%) at 5 years (Yu et al., 2010; Perez Horta et al., 2016; Yu et al., 2021). These results led to its marketing authorization by the FDA in the United States of America in 2015. In 2017, the European Commission granted marketing authorization of dinutuximab ß, a ch14.18 mAb (expressed in CHO cells instead of SP2O cells) after a phase III clinical trial conducted by the SIOPEN (Ladenstein et al., 2018).

The murine 3F8 was similarly humanized by fusing complementarity-determining regions to a human IgG1 framework, thus forming hu3F8. The hu3F8 maintained affinity of the murine antibody, but increased ADCC capacity and drastically lowered CDC capacities (Cheung et al., 2012). In a pilot Phase I trial of hu3F8 on resistant neuroblastoma, 79% of patient treated with highest acceptable dosage had partial or complete response (Kushner et al., 2018). This led in 2020 to accelerated approval of hu3F8 in relapsed/refractory neuroblastoma patients (Markham, 2021), increasing the panel of approved anti-GD2 therapies available to patients. Acute pain during antibody infusion and occasional neuropathy were observed but with no late toxicities reported in long-term follow-up of patients treated with anti-GD2 mAbs (Ozkaynak et al., 2000; Yu et al., 2010; Kushner et al., 2011; Ladenstein et al., 2018).

**FIGURE 3:**
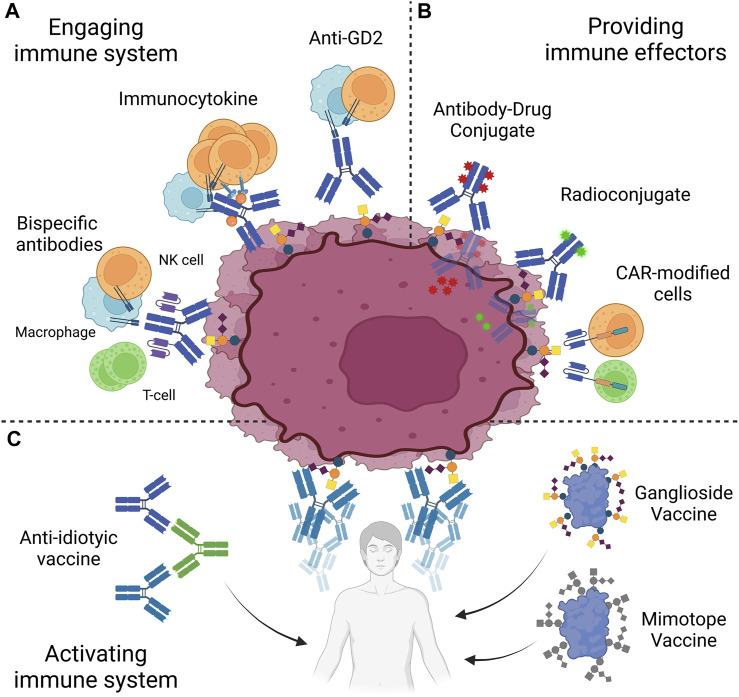
Anti-GD2 immunotherapies. Strategies to target GD2 are based on three approaches. **(A)** Patient immune system is engaged against GD2-positive cells through antibodies, immunoctykines or bispecific antibodies. **(B)** Immune effectors, such as antibody-drug conjugates, radioconjugates, or CAR-modified cells, are directly provided to the patient. **(C)** Patient immune system may be activated to target GD2 through the use of vaccines, based on purified GD2, peptide mimotopes or anti-idiotypes. NK, Natural Killer; CAR, Chimeric Antigen Receptor. Created with BioRender.com.

Clinical success of anti-GD2 antibodies demonstrated the potential of anti-GD2 immunotherapies. However, their clinical use is hindered by neurologic adverse effects of on-target off-tumor nature. Moreover, a number of patients remain refractory or relapse, even in GD2-positive cancers. In this context, several strategies to optimize success of therapies will be discussed here. Avenues to optimize anti-GD2 treatment rely either on increasing the administrable dose of antibodies, limited by the side effects, or exploring other immunotherapeutic mechanisms (Figure 3).

### 8.2 Necessity of companion diagnostics

As stated earlier, GD2 expression is heterogeneous both between and within tumors, which could pose an obstacle for effective tumor targeting. GD2 is heterogeneously expressed in various cancers, and may as such hold diagnostic and prognostic value, whether expressed on tissues, or shed as was mentioned in the context of cancer previously. Conflicting reports undermine GD2-expression’s prognostic relevance (Valentino et al., 1990; Czaplicki et al., 2009; Balis et al., 2020; Erber et al., 2021; Galan et al., 2023)**.** However, shed or tumor-expressed GD2 may be used as a companion diagnostic for anti-GD2 immunotherapies, particularly in cancers where heterogeneous expression has been identified (See Table 1) (Portoukalian et al., 1993; Wiebel et al., 2021)**.** In addition, GD2 may be used as a diagnostic tool. Studies have shown that serum levels of GD2 separate between patients with NB and healthy controls (Schulz et al., 1984). Serum level of GD2 of patients with ovarian cancers was reported to hold higher diagnostic power than standard CA125 detection for the screening of ovarian cancer of all types (Galan et al., 2023). In this context, the challenge lies in developing clinically applicable routine assays, due to GD2 biochemical nature and the absence of reference controls. Initially, detection of circulating/shed GD2 was conducted after ganglioside extraction, followed by chromatographic separation using high-performance thin layer chromatography and densitometric scanning (Ladisch et al., 1987; Valentino et al., 1990; Portoukalian et al., 1993). Since then, different methods have emerged to detect circulating GD2. A more recent standard for GD2 detection and quantification is the use of high-performance liquid chromatography coupled to mass spectrometry (Busch et al., 2018; Balis et al., 2020). This technique enables quantification of gangliosides with precision as low as 4–6 ng/mL, and separation of GD2 subspecies based on long chain bases. In a simplified technique, ganglioside stripped of their ceramide and fluorescently labeled were quantified using HPLC only through the differential retention time of their glycan moiety, producing results comparable to HP-TLC (Czaplicki et al., 2009)**.** In a recent report, a quantitative ELISA was performed on serum samples and displayed similar sensitivity as HPLC-MS for GD2 when using a purified standard (Galan et al., 2023). Circulating GD2 may additionally be detected indirectly. A surprising report suggests that the epitope detection in monocytes (EDIM) blood test utilizing GD2 could function as a sensitive and non-invasive diagnostic tool for detecting tumor cells in patients with NB (Stagno et al., 2022). EDIM is based on antigen expression by macrophages which phagocytose parts of neoplastic cells, extracellular vesicles, or circulating tumor cells, and subsequent identification by flow cytometry in blood of patients (Stagno et al., 2022). Further studies on this method are, however, required before it can be widely adopted as a standard diagnostic approach**.** Apart from circulation, GD2 expression may also be detected on tumor cells. This aspect has particular relevance in therapeutic decisions.

The current gold standard for tissue expression of GD2 is immunohistochemistry, which can be conducted on primary tumors of bone marrow aspirates (Schulz et al., 1984; Berthold et al., 1989; Sariola et al., 1991; Chang et al., 1992; Corrias et al., 2008).

In the context of residual disease such as bone marrow infiltration, quantification of B4GALNT1 (GM2/GD2S) via RT-qPCR similarly showed high sensitivity (Hoon et al., 2001; Lo Piccolo et al., 2001; Cheung et al., 2003; Laurent et al., 2010). One report comparing both methods in this context revealed higher sensitivity of RT-qPCR quantification of GD2S than immunohistochemistry (Lo Piccolo et al., 2001). RT-qPCR however possesses the caveat of not quantifying strictly GD2 levels, as GM2 may instead be upregulated by this same enzyme. These two techniques may then be used for different purposes, with immunohistochemistry revealing pertinence of anti-GD2 therapy in a patient, and B4GALNT1 quantification used for precise monitoring of remission.

### 8.3 Engaging the patient’s immune system

As mentioned earlier, a common acute side effect upon anti-GD2 mAb infusions is severe pain that limits the administrable dose to patients (Ozkaynak et al., 2000; Yu et al., 2010; Kushner et al., 2011; Tong et al., 2015; Ladenstein et al., 2018)**.** This neuropathic pain has been attributed to complement activation on anti-GD2 bound peripheral nerves (Sorkin et al., 2010). To reduce this side effect, a single point mutation (K322A) was introduced in the Fc receptor of the humanized 14.18 anti-GD2 mAb to decrease C1q binding while maintaining ADCC capabilities (Sorkin et al., 2010). The resulting antibody hu14.18K322A elicited less allodynia in rats than dinutuximab (Sorkin et al., 2010). At the clinical level, this enabled higher antibody dosage (Anghelescu et al., 2015), and the result of a phase II clinical trial showed that usage of hu14.18K322A displays similar efficacy with reduced pain in patients with NB (Furman et al., 2022). An alternative approach to mitigate severe pain associated with anti-GD2 treatment involves targeting OAcGD2, expressed on GD2-positive tumors but not on peripheral nerves (Alvarez-Rueda et al., 2011). The mAb 8B6, specifically targeting OAcGD2, demonstrated comparable immune-mediated tumor-killing effects *in vitro* and inhibition of tumor growth *in vivo* to anti-GD2 antibody, without inducing allodynia in rats ((Alvarez-Rueda et al., 2011; Terme et al., 2014). Clinical trials are anticipated to explore optimal dosing of anti-OAcGD2 mAb.

To potentiate antibody-based treatment, strategies to increase patient immune cells’ engagement may be devised. Based on the anti-GD2 antibodies partial reliance on immune effectors such as NK cells and macrophages, immunocytokines were developed. Immunocytokines are conjugate between an antibody and cytokines, thus limiting their systemic effect, while bolstering activity and proliferation of tumor microenvironment immune cells (Neri and Sondel, 2016). The anti-GD2 hu14.18 antibody expressed as a fusion protein with interleukin-2 (hu14.18-IL2) has shown promise in treating NB during preclinical and clinical testing. In a Phase II study of this therapeutic on relapsed or refractory neuroblastoma, a fifth of 23 patients responded to treatments, all displaying complete response from 9 to 35 months (Shusterman et al., 2010). However, doubts remain regarding the overall therapeutic benefits of IL-2 due to the severe toxicities associated with higher doses and the unestablished effectiveness at lower doses (Shusterman et al., 2010). Another potentially useful cytokine to bolster the patient’s immune system is IL-15 (Vincent et al., 2013). Interestingly, in a preclinical study, hu14.18-IL-15 & IL-21 outperformed hu14.18-IL2 in immunocompetent mice with syngeneic NB with increased survival (Nguyen et al., 2022). The increase in survival was concurrent with increased tumor CD8^+^ T cells and M1 macrophages, and decreased Treg. Interestingly this increased recruitment was mediated by the CXCL9/10 axis, seen to be activated during decreased lipid raft formation caused by overexpression of GD1S, which could similarly apply to antibody incubation, thus producing a synergistic effect (Nguyen et al., 2022).

Anti-GD2 monoclonal antibodies have also paved the way for the development of bi- and tri-specific antibodies. These novel antibody constructs redirect T-cell cytotoxicity towards GD2-positive cells by linking tumoral antigens and T-cell receptors via anti-CD3 (Cheng et al., 2015; Cheng et al., 2016). Bispecific antibodies, such as huOKT3-hu3F8, have demonstrated potent tumor control (Cheng et al., 2016). Trispecific antibodies, formed by linking ScFv to anti-GD2 IgG’s light chains, not only redirect T-cells but also engage Fc-receptor bearing immune cells. In murine models, trispecific antibodies based on ch14.18 or hu3F8 exhibited remarkable control over metastatic neuroblastoma, melanoma, and osteosarcoma tumors (Xu et al., 2015; Park and Cheung, 2020; Nakajima et al., 2021; Park et al., 2021; Zirngibl et al., 2021). Encouraged by preclinical success, phase I/II clinical trials employing trispecific antibodies (hu3F8) have been initiated for neuroblastoma, osteosarcoma, and small cell lung cancer (Hattinger et al., 2019; Yankelevich et al., 2019; Ordóñez-Reyes et al., 2022). Initial results from the neuroblastoma and osteosarcoma trials indicate a 33% clinical response rate among patients with relapsed/refractory disease who completed Phase I (Yankelevich et al., 2019). Phase II results will provide further insights into the effectiveness of this therapeutic approach.

### 8.4 Providing immune effectors

Another strategy is to directly provide cytotoxic effectors redirected via antigen specific whole or fragment antibody. Radiolabeled antibodies, enables targeted therapy combining diagnostic and therapeutic capabilities (aka theranostics). A pilot study using ^131^I-GD2 ch14.18 in patients with NB demonstrated tumor uptake in 65% of cases with acceptable organ doses (Zhang et al., 2022). A Phase II study on medulloblastoma using ^131^I-3F8 resulted in 15 long-term survivors out of 42 patients, showing promising outcomes (Kramer et al., 2018). The Sloan Kettering Cancer Center (New York City, NY, United States) adopted ^131^I-labeled anti-GD2 3F8 as a standard protocol for patients with high-risk NB older than 1 year, starting from reference protocol N7 (Cheung et al., 2001). Of note, imaging with ch14.18-labeled ^99m^Tc improved NB recurrence detection (Reuland et al., 2001). In small animal bearing NB tumors, gold particles conjugated to anti-GD2 hu14.18K322A showed enhanced CT contrast imaging, while exhibiting an increased NK cell cytotoxicity against GD2-positive tumors compared to that elicited by unlabeled hu14.18K322A. Conjugation of anti-GD2 to gold particles may additionally enable photo-thermolysis of GD2-positive cells following near-infrared laser light exposure (Peng and Wang, 2011).

Building up on data showing internalization of the 14G2a antibody, various payloads were conjugated to anti-GD2 (Kalinovsky et al., 2022; Tibbetts et al., 2022). Surprisingly, there is a scarcity of studies on classical antibody-drug conjugates based on anti-GD2. Recently, conjugation of ch14.18 with the microtubular poisons monomethyl auristatin E & F was achieved via a cleavable linker to release the payload inside target cells after endocytosis. Both antibody drug conjugates achieved superior tumor control compared to the parent ch14.18 antibody, with significant differences in mice GD2-positive melanoma and lymphoma tumor volume. This opens the way for further work based on this model, exploiting the full diversity of drug-conjugates already in clinical trials (Fu et al., 2022). Of note, hydrophobic drugs may be used in anti-GD2-linked liposomes and delivered effectively (Di Paolo et al., 2011; Jose et al., 2020).

The anti-GD2 14G2a and 3F8 mAbs have also been used to develop chimeric antigen receptors expressed particularly on αβ T cells. CARs consist of a cell surface antigen-specific single-chain variable fragment (ScFv) linked to signaling domains. Co-stimulatory domains, such as CD28, CD137, and/or OX40, are utilized in CAR-T cells, linked to the CD3ζ signaling domain. Clinical trials using GD2 CAR-T cells have been conducted, primarily in relapsed/refractory neuroblastoma (Louis et al., 2011; Heczey et al., 2017). Third-generation CAR-T cells using a 14G2a ScFv showed promising results, with complete remission achieved in a subset of patients (Del Bufalo et al., 2023). A Phase I/II trial using a hu3F8-based ScFv on a fourth-generation CAR demonstrated a 1-year overall survival rate of 74% and partial responses in a portion of patients (Yang et al., 2017; Yu et al., 2022). A comparison of these trials highlighted that increased affinity and the use of a fourth-generation construct did not necessarily result in superior effects. GD2 CAR-T cells have also been tested in glioblastoma and H3K27M-mutated diffuse intrinsic midline gliomas, showing clinical improvements in some patients (Majzner et al., 2022).

CAR-T cells are not the only cell types modified with CARs for GD2-positive cancers. Expression of GD2-specific CAR on macrophages and mesenchymal cells led to GD2-positive tumor cell cytotoxicity, proving potential utility, although their naturally pro-tumorigenic role in the tumor microenvironment remains to be addressed (Golinelli et al., 2022; Golinelli et al., 2020; Zhang et al., 2023). NK cells and γδ T cells, which play crucial roles in antitumor immunity and are found infiltrated in tumors, have also been modified with CARs, resulting in GD2-positive cancer cell lysis (Capsomidis et al., 2018; Chabab et al., 2020; Cózar et al., 2021; Zuo et al., 2023). However, these cell types have a shorter lifespan and memory cell formation remains uncertain compared to αβ T cells (Sun et al., 2011; Comeau et al., 2020). NKT cells, semi-innate lymphoid cells sharing properties of both NK cells and T cells, have shown efficacy in preclinical studies, and a Phase I trial using CAR GD2 iNKT cells derived from the 14G2a antibody was initiated for neuroblastoma patients, showing potential with complete and partial remissions observed (Heczey et al., 2014; Xu et al., 2019). Overall, CAR-based immunotherapies targeting GD2 have demonstrated promising results in various cancers, including neuroblastoma and glioblastoma, with manageable safety profiles. The utilization of different cell types and CAR designs provides a range of options for personalized treatment approaches.

### 8.5 Activating the patient’s immune system

A final strategy is complete reliance on the patient’s immune system activation, named active immunotherapy. In the case of GD2, a glycolipid with poor immunogenicity, vaccine development has been the primary focus. GD2’s glycan portion, which constitutes its hydrophilic extracellular membrane, poses a challenge for vaccine development since carbohydrate antigens are not strongly immunogenic. To enhance immunogenicity, GD2 has been conjugated to carrier proteins like keyhole limpet hemocyanin (KLH) and combined with adjuvants. Initial attempts using GD2-KLH and Monophosphoryl-Lipid A (MPLA) adjuvant failed to induce anti-GD2 antibodies in glioma patients. However, GD2-lactones (GD2L) conjugated to KLH with QS-21 adjuvant successfully elicited anti-GD2 antibodies in over 80% of melanoma patients, with responses lasting more than 6 months (Nores et al., 1987; Ragupathi et al., 2003). Vaccine trials involving mixtures of ganglioside conjugates such as GD2L and GD3L-KLH with OPT-821 and ß-glucan demonstrated encouraging results in neuroblastoma, leading to high anti-GD2 titers and more than 90% long-term survival rates (Kushner et al., 2014; Cheung et al., 2021). A bivalent vaccine targeting GM2 and GD2-KLH induced both anti-GM2 and anti-GD2 responses in nearly half of melanoma patients (Chapman et al., 2000). However, a trivalent vaccine combining GM2, GD3L, and GD2L-KLH with OPT-821 showed no advantage in overall or progression-free survival compared to adjuvant alone in a phase II study on relapsed sarcoma patients (Rosenbaum et al., 2022). This suggests that the addition of ß-glucan adjuvant may have been beneficial. The context and selection of ganglioside vaccines are critical factors to consider for the success of this approach.

In order to circumvent the difficulties linked to the use of purified GD2 gangliosides, approaches were developed to produce mimics. Mimotopes are peptide sequences developed to imitate molecules. In the case of GD2, phage display technology was used to raise peptides against the ch14.18 antibody, aiming to produce structural copies of GD2 (Förster-Waldl et al., 2005; Fest et al., 2006; Riemer et al., 2006)**.** Mimotopes could be delivered *in vivo* directly, conjugated to KLH to increase immunogenicity, or through DNA vaccines with adjuvant (Fest et al., 2006; Riemer et al., 2006). In both cases, this induced a humoral response, capable of clearing liver metastases with NK activation with the DNA vaccine. Interestingly, DNA vaccines expressing mimotopes based on the 14G2a antibody led to production of antibodies reactive against GD2 and NCAM, just as 14G2a (Bolesta et al., 2005). One mimotope vaccine mimicking both GD2 and Lewis Y antigen, conjugated to a pan T-cell epitope and with adjuvant, was used in a Phase I trial in stage IV breast cancer patients (Hutchins et al., 2017). Encouragingly, this led to production of antigens to both antigens in all patients, and may pave the way for further studies.

Another technique mimicking GD2 is the development of anti-idiotype vaccines. An anti-idiotype is an antibody raised against the variable region of another antibody, thus adopting a similar conformation to the epitope of this second antibody. This theory was applied to GD2 after the observation of anti-idiotype (anti-Id) antibodies in melanoma patients following treatment with the murine 14G2a (Saleh et al., 1993). By immunizing rodents with either 3F8 or 14G2a, anti-Id antibodies were generated, capable of inhibiting anti-GD2 binding to GD2-positive targets. Furthermore, immunization of allogenic mice with these anti-Id led to *in vivo* production of anti-GD2 (Cheung et al., 1993; Sen et al., 1998)**.** Similar results were observed in primates using 1A7, the anti-Id raised against 14G2a, with QS-21 adjuvant, leading to clinical evaluation of 1A7 (Sen et al., 1997). Phase I clinical trials were conducted on patients with advanced melanoma. In these trials, 85% of patients developed an anti-GD2 response with 1 complete response and 12 stable diseases on 47 patients going for 1 + year (Foon et al., 1998; Foon et al., 2000)**.** In contrast, in high-risk neuroblastoma following complete remission, all patients developed anti-GD2, and 85% of patients in first remission remained without disease progression, compared to 10% for patients in second remission (Batova et al., 2004). More recently, another anti-Id, named ganglidiomab was raised in mice against 14G2a, and further humanized to produce the chimeric ganglidiximab (Lode et al., 2013; Eger et al., 2016). Ganglidiximab was capable of raising anti-GD2 responses, and may increase the specificity of this response, enhancing effect of this therapeutic against tumoral challenges.

Interestingly, GD2 carbohydrate head-groups displayed on a multivalent polyamidoamine scaffold used as vaccines induced rapid expansion of γδ T cells in mice, followed by a second-wave CD8^+^ T cells expansion (Bartish et al., 2020).

Intriguingly, a role for GD2 has a target for un-engineered cytotoxic T cells was described in mice. Immunization of mice with a GD2 expressing cell line induced a cytotoxic T cell response, carried out by CD8^+^ T cell (Zhao and Cheung, 1995). The cytotoxicity was dependent on the TCR, independent of NK cells, and restricted to GD2 and H-2b bearing targets. Functionally, it was suggested that GD2 residues could be linked to peptides, working as haptens, that bind to the MHC class I pocket. Pending confirmation, this could pave the way for alternative targeting of GD2-positive cancers, such as vaccine therapy and TCR engineering.

## 9 Conclusion and perspectives

While encouraging results have been observed using different therapeutic modalities, cases of refractory or relapsed disease are still frequent. Thus, the different treatment may benefit from potentiation based on biological properties of GD2. Based on monoclonal antibodies’ inhibition of the FAK/AKT/mTOR pathway in GD2-positive tumors, inhibition of components of the pathway or others involved in cell survival may achieve targeted and synergistic effect, thus enhancing therapeutic effect. Building up on GD2’s role as an immune checkpoint for macrophages, synergistic use of anti-GD2 and anti-CD47—a phagocytosis suppressor–has been used to attain synergy and eradicate *in vivo* osteosarcoma and small cell lung cancer tumors (Ito et al., 2001; Theruvath et al., 2022).

The potential expression of GD2 on T-cell warrants particular attention in T-cell based immunotherapies, as it could potentially lead to fratricidal killing and drop in therapeutic efficacy, as seen in other contexts (Kozani et al., 2021). The same may apply with CAR-macrophages since after tumor cell phagocytosis they may recycle the tumor cell GD2 on their cellular membrane (Stagno et al., 2022). Immune effector cell candidates for CAR approaches should be tested respectively against the immunosuppressive properties of tumor cell shed GD2. A different approach, based on GD2’s propension to be shed from tumor cells, is the inhibition of extravesicular vesicles’ secretion has proven effective in a preclinical model (Liu et al., 2022). While this might inhibit transfer of malignant properties from GD2-positive to antigen-negative cells, it especially potentiated anti-GD2’s therapeutic effect through reduction of decoy targets and immunosuppressive properties of circulating GD2. Another strategy will be the use of anti-GD2 immunotherapies at the time of minimal residual disease.

Regarding the development of vaccines, different alternatives can be suggested to further improve efficacy. As mentioned, the choice of GD2 subspecies may matter, with varying immunogenicity among the different structures, as seen with increased efficacy using lactonized forms of GD2 (Nores et al., 1987). In addition, the potential of GD2 stabilizing peptides to induce cytotoxic T-cell response remains to be explored.

Finally, a commonality between all treatments is the superior results obtained in the treatment of high-risk neuroblastoma compared to other indications. One specificity of neuroblastoma is high expression of GD2, observable in nearly all cells, with very rare cases of antigen loss (Wu et al., 1986; Kramer et al., 2001). As such, other GD2-positive cancer may benefit from strategies to increase antigen expression to levels similar to neuroblastoma. Different compounds may achieve this effect, such as epigenetic modulators (HDAC or EZH2 inhibitors), cytokines (IFNγ, TNFα, IL-4) which may additionally potentiate immune effectors, retinoids (Fenretinide), or polyamine (nanospermidine) as more recently described (Hoon et al., 1991a; Hoon et al., 1991b; Shibina et al., 2013; Kroesen et al., 2016; van den Bijgaart et al., 2019; Kailayangiri et al., 2019; Mabe et al., 2022; Galassi et al., 2023). Identifying the most suitable patients and the optimal timing for these approaches should also be taken into consideration to improve the patients’ outcome. In this regard, the quantification of tumor shed GD2 can provide a diagnostic companion for anti-GD2 immunotherapies.
